# Correcting dynamic distortions in 7T echo planar imaging using a jittered echo time sequence

**DOI:** 10.1002/mrm.26018

**Published:** 2015-11-19

**Authors:** Barbara Dymerska, Benedikt A. Poser, Wolfgang Bogner, Eelke Visser, Korbinian Eckstein, Pedro Cardoso, Markus Barth, Siegfried Trattnig, Simon D. Robinson

**Affiliations:** ^1^ High Field MR Centre, Department of Biomedical Imaging and Image‐Guided Therapy, Medical University of ViennaViennaAustria; ^2^ Facultyof Psychology and Neuroscience, Department of Cognitive Neuroscience, Maastricht UniversityMaastrichtNetherlands; ^3^ FMRIB Centre, Nuffield Department of Clinical Neurosciences, University of OxfordOxfordUnited Kingdom; ^4^ Centre for Advanced Imaging, University of QueenslandBrisbaneQueenslandAustralia

**Keywords:** field mapping, dynamic distortion correction, ultra‐high field, EPI, fMRI, respiration effects

## Abstract

**Purpose:**

To develop a distortion correction method for echo planar imaging (EPI) that is able to measure dynamic changes in B_0_.

**Theory and Methods:**

The approach we propose is based on single‐echo EPI with a jittering of the echo time between two values for alternate time points. Field maps are calculated between phase images from adjacent volumes and are used to remove distortion from corresponding magnitude images. The performance of our approach was optimized using an analytical model and by comparison with field maps from dual‐echo EPI. The method was tested in functional MRI experiments at 7T with motor tasks and compared with the conventional static approach.

**Results:**

Unwarping using our method was accurate even for head rotations up to 8.2°, where the static approach introduced errors up to 8.2 mm. Jittering the echo time between 19 and 25 ms had no measurable effect on blood oxygenation level–dependent (BOLD) sensitivity. Our approach reduced the distortions in activated regions to <1 mm and repositioned active voxels correctly.

**Conclusion:**

This method yields accurate distortion correction in the presence of motion. No reduction in BOLD sensitivity was observed. As such, it is suitable for application in a wide range of functional MRI experiments. Magn Reson Med 76:1388–1399, 2016. © 2015 The Authors Magnetic Resonance in Medicine published by Wiley Periodicals, Inc. on behalf of International Society for Magnetic Resonance in Medicine. This is an open access article under the terms of the Creative Commons Attribution License, which permits use, distribution and reproduction in any medium, provided the original work is properly cited.

## INTRODUCTION

Gradient‐recalled echo planar imaging (EPI) is the most commonly used sequence for functional MRI, providing high sensitivity to blood oxygenation level–dependent (BOLD) signal changes and high temporal resolution. BOLD sensitivity is increased by the use of ultrahigh magnetic fields 1, 2, 3, 4. EPI at ultrahigh field suffers, however, from increased geometric distortions 5, particularly near the ear canals and sinuses. Distortion also changes dynamically due to head motion 6, 7 and respiration 8, 9, 10, 11. Both static and dynamic effects lead to mislocalization of activation. The most common approach to correct distortion is to measure a single B_0_ field map (FM) and use this to unwarp all EPI volumes in subsequent functional MRI (fMRI) runs (called static distortion correction [SDC]) 12, 13, 14. A single FM does not capture motion and respiration related effects, however. It is therefore desirable to develop a dynamic distortion correction (DDC) method in which an up‐to‐date FM is generated and applied to each volume.

Dynamic distortion correction can be realized using FMs calculated from multiecho EPI 15 phase data (an approach known as Dynamic Off‐resonance Correction with Multiecho Acquisition [DOCMA]) 16. The acquisition of at least two echoes, however, imposes spatio‐temporal limitations, particularly with short T2* values at ultrahigh magnetic fields.

Several DDC approaches based on single‐echo EPI have been proposed 17, 18, 19, 20. All of them share the assumption that the echo time (TE)–independent phase contribution, arising primarily from B1 field inhomogeneities and often termed the “phase offset” ( $\varphi_{o}$) is constant throughout the time series. Subtracting $\varphi_{o}$ from the phase images and dividing by the echo time (TE) yields a FM 17, 18. DDC methods in this category need a reference scan in order to fully unwarp EPI, and have only been tested for moderate motion [eg, up to 0.2° rotation 18] using volume coils 18, 20 and at field strengths up to 3T 17, 18, 19, 20, where $\varphi_{o}$ varies slowly in space. For measurements at 7T with multichannel coils, each coil element is subject to a different offset, and the RF wavelength is shorter 21. The assumption of temporal stability of $\varphi_{o}$ has yet to be examined, especially for larger motion and multichannel coils.

We propose a reference‐free approach in which the TE is “jittered” between two values, one for odd and one for even time points, without loss of temporal resolution. FMs are calculated from consecutive pairs of volumes and used to correct distortion in corresponding magnitude images. We refer to this method as jittered‐TE DDC. The effects of motion and respiration on the accuracy of jittered‐TE DDC are studied analytically and experimentally. The performance of the jittered‐TE approach is assessed with respect to a reference dual‐echo method, DOCMA, and in comparison with SDC. The BOLD sensitivity of the jittered‐TE sequence is compared with that of standard EPI in a block design experiment incorporating hand and foot tasks. Finally, we compare the accuracy of SDC and jittered‐TE DDC in the localization of activation in the primary motor cortex.

## THEORY

The local magnetic field inhomogeneity (ΔB_0_, in Hz) is proportional to the difference between two phase images, Δφ, obtained at different echo times ( ${TE}_{1}$, ${TE}_{2}$) 12:

(1) $$\Delta B_{0}\left(x,y,z\right)=\frac{\varphi_{{TE}_{2}}\left(x,y,z\right)-\varphi_{{TE}_{1}}\left(x,y,z\right)}{2\pi ({TE}_{2}-{TE}_{1})}=\frac{\Delta \varphi \left(x,y,z\right)}{2\pi \Delta TE}.$$

All calculations are performed voxel‐wise, and the $\left(x,y,z\right)$ coordinates are omitted in further expressions.

A voxel shift map (VSM), specifying how much each voxel has to be shifted in order to restore the signal to the correct position, can be derived from ΔB_0_:

(2) $$VSM=\Delta B_{0}\cdot t_{etl},$$

where $t_{etl}$ is the total echo train length.

The phase images $\varphi_{{TE}_{1}}$ and $\varphi_{{TE}_{2}}$ are commonly acquired using a gradient echo (GE) sequence prior to the EPI time series, and yielding a single static FM which is applied to subsequent EPI volumes in one or more time series (or “runs”). In the DOCMA approach, $\varphi_{{TE}_{1}}$ and $\varphi_{{TE}_{2}}$ are obtained from two echoes of a multiecho EPI sequence, and ΔB_0_ is calculated for each time point.

The jittered‐TE approach is based on a single‐echo EPI with a jitter of the echo time (ie, one TE for odd and another for even time points). ΔB_0_ can be calculated from the phase evolution between each odd‐even pair of volumes. This should represent field inhomogeneities at both time points, but the FM can be disrupted by changes in frequency between consecutive time points due to respiration 10, motion, or scanner instabilities. To assess this effect, we express the frequencies as $\omega_{2}=\omega_{1}+\delta \omega$, where δω is a frequency change between time point 1 and 2. The corresponding phases are $\varphi_{1}={TE}_{1}\cdot 2\pi \omega_{1}$ and $\varphi_{2}={TE}_{2}\cdot 2\pi \left(\omega_{1}+\delta \omega \right)$ if ω=const during each echo acquisition. Substituting $\varphi_{1}$ and $\varphi_{2}$ in Equation (1) by the above expressions yields

(3) $$\Delta B_{0}=\frac{{TE}_{2}\cdot \left(\omega_{1}+\delta \omega \right)-{TE}_{1}\cdot \omega_{1}}{\left({TE}_{2}-{TE}_{1}\right)}=\omega_{1}+\delta \omega \left(\frac{{TE}_{1}}{\Delta TE}+1\right)=\omega_{2}+\delta \omega \left(\frac{{TE}_{2}}{\Delta TE}-1\right),$$

making it apparent that ΔB_0_ deviates from the true values by $\delta \omega \left({TE}_{1}/\Delta TE+1\right)$ for the first time point and $\delta \omega \left({TE}_{2}/\Delta TE-1\right)$ for the second time point. These errors can therefore be reduced by decreasing TE or increasing ΔTE. Additionally, in the sampling regime below the Nyquist rate, decreasing repetition time (TR) also reduces errors, because faster sampling reduces δω.

## METHODS

### Image Acquisition

Measurements were performed with a 7T whole body MR Magnetom scanner (Siemens Healthcare, Erlangen, Germany) and a 32‐channel head coil (Nova Medical, Wilmington, Massachusetts, USA). The study was approved by the Ethics Committee of the Medical University of Vienna. Eleven volunteers (men, n = 9; women, n = 2; age, 27 ± 4 years) participated in the study after providing written, informed consent. Four experiments were designed to estimate FM errors, optimize the jittered‐TE sequence, and evaluate the performance of the approach in the presence of large motion and in a block design fMRI experiment. High temporal but low spatial resolution EPI was acquired with the birdcage transceive coil for experiment 1 to capture the dynamics of respiration‐related phase changes. The 32‐channel array was used in all other experiments. A combined dual‐echo jittered‐TE sequence was used in experiment 2 so that the jittered‐TE could be optimized and both the jittered‐TE and SDC methods could be compared with the reference DOCMA method. A single‐echo jittered‐TE sequence was used for experiments 3 and 4 to avoid the spatio‐temporal and TE constraints of multiecho acquisitions. All EPIs were measured with a posterior–anterior phase encoding direction to reduce signal pile‐up. Dual‐echo GE scans were also acquired for SDC: two before and two after each EPI run. These had the same geometry as the EPI and TEs = [5,10] ms. The readout direction was reversed in the second scan of each pair to allow gradient delay effects to be eliminated 22.

#### Experiment 1: Estimation of Respiration‐Induced Phase Fluctuations

Seven volunteers were instructed to lie still and breathe normally. Phase images were acquired using single‐echo EPI with a low spatial and high temporal resolution and the following parameters: matrix = 64 × 64; 9 slices with 10% gap; voxel dimensions = 3.3 × 3.3 × 11.2 mm^3^; flip angle (FA) = 40°; receiver bandwidth (RBW) = 1447 Hz/pixel and 6/8 partial Fourier; TR = 520 ms; TE = 22 ms; and 120 volumes. Respiration was recorded using a chest belt.

#### Experiment 2: Quantification of Respiration and Motion‐Induced Field Mapping Errors and Optimization of the Jittered‐TE Sequence

Four volunteers were asked, in a first acquisition, to lie still and, in a second acquisition, to rotate their head slowly about the left–right axis (ie, a slow nod). A dual‐echo jittered‐TE sequence was implemented to allow estimation of the accuracy of the jittered‐TE approach with respect to the reference DOCMA method with different ΔTEs and TRs. The parameters of this combined sequence were adjusted within the spatio‐temporal constraints of a dual‐echo acquisition using the following parameters: matrix = 64 × 64; 9 slices with 10% gap; voxel dimensions = 3.3 × 3.3 × 4.4 mm^3^; FA = 58°; RBW = 1474 Hz/pixel; GRAPPA factor 2 and 6/8 partial Fourier; TE_odd_ = [11,31] ms and TE_even_ = [11 + ΔTE, 31 + ΔTE] ms, with ΔTE = [0.8,2,4,6] ms; TR = 1200 ms; and 50 volumes. An additional measurement was made with ΔTE = 6 ms, TR = 2400 ms, and 25 volumes.

#### Experiment 3: Comparison of the Accuracy of the Static and Jittered‐TE Dynamic Distortion Correction in the Presence of Motion

Three volunteers performed a slow nod during the jittered‐TE EPI scan. Other than the jittering of the echo time, sequence parameters were typical for whole‐brain EPI fMRI at 7T 23, 24: matrix = 128 × 128; 38 slices with 20% gap; voxel dimensions = 1.64 × 1.64 × 2.0 mm^3^; FA = 58°; RBW = 1447 Hz/pixel; GRAPPA factor 2 and 6/8 partial Fourier; TR = 2400 ms; and 25 volumes. The odd/even TEs were centered on 22 ms with ΔTE = 6 ms (ie, TE_odd,even_ = [19,25] ms).

#### Experiment 4: Evaluation of the Performance of Jittered‐TE DDC in a Block Design fMRI Task

Jittered‐TE EPI (with the same parameters as in experiment 3) was compared with a standard single echo EPI (TE = 22 ms) in a motor task. Six volunteers were asked to perform hand clench‐release and foot dorsi‐plantar flexion with the dominant hand and foot with an approximate frequency of 2 Hz in the following block design: A = rest, B = hand clench‐release, C = foot flex in an ABACABACA pattern with 16 volumes per block (block duration 19.2 s) and a total of 72 volumes. Six runs were acquired per volunteer, three with each sequence (jittered‐TE and standard EPI), in an interleaved fashion.

### Data Analysis

Separate channel magnitude and phase images were postprocessed in MATLAB (MathWorks, Natick, Massachusetts, USA). Phase differences were obtained using a separate channel approach 25 for GE‐based FMs and a Hermitian inner product 26 for EPI‐based (DOCMA and jittered‐TE) FMs. Phase unwrapping was performed using 2D PRELUDE v2.0 27. Residual phase jumps of integer multiples of 2π between adjacent slices and time points were removed 21. FMs were calculated from phase difference images according to Equation (1), and VSMs were derived using Equation (2). For static FM, two field maps with opposite readout polarities were averaged, removing gradient delay effects.

#### Experiment 1: Estimation of Respiration‐Induced Phase Fluctuations

Regions of interest (ROIs) with 3 × 3 voxels positioned centrally (but avoiding CSF) were selected manually in unwrapped and jump‐corrected phase in all brain slices. The mean and standard deviation of the phase difference between respiration minima and maxima were calculated for each ROI in each slice. The dominant frequency in the spectrum of phase fluctuations was noted as the respiration frequency of that volunteer.

#### Experiment 2: Quantification of Respiration and Motion‐Induced Field Mapping Errors and Optimization of the Jittered‐TE Sequence

**Figure 1:**
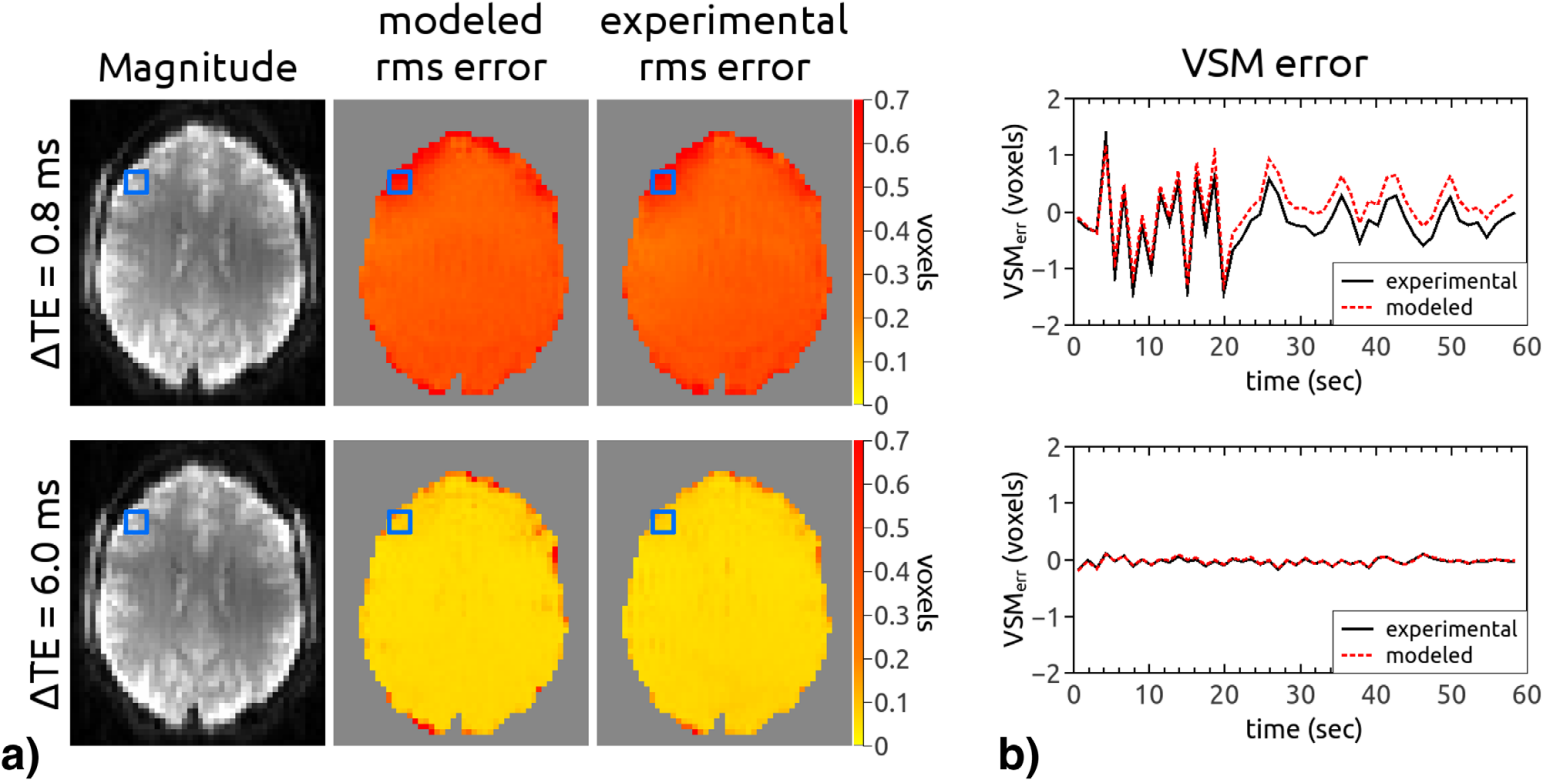
Comparison between jittered‐TE VSM error maps obtained from the model and those calculated experimentally for a representative subject. Data are taken from scans with ΔTE = 0.8 and 6.0 ms and no intentional motion. (**a**) Root‐mean‐square error maps and corresponding magnitude images to indicate slice location. (**b**) Jittered‐TE VSM errors in selected ROIs (blue squares in panel a) as a function of time. Both the error maps and the plots show that the model accurately estimates the experimental errors in jittered‐TE field mapping.

DOCMA FMs were calculated from the dual‐echo data at each time point. Jittered‐TE FMs were calculated from pairs of consecutive volumes using the first echo of each time point (TE_odd,even_ = [11,11 + ΔTE] ms). All FMs were transformed into VSMs to represent FM errors in more intuitive units. The voxel‐wise difference between 1) static and DOCMA as well as 2) jittered‐TE and DOCMA VSMs was calculated and, for a selected ROI, plotted as a function of time (Fig. 1b). Root‐mean‐square error maps were calculated from VSM differences: $E_{rms}=\surd (\sum_{N}{VSM}_{diff}^{2})/N$, where N is the total number of volumes (Fig. 1a). Additionally, a comparison between experimental and modeled jittered‐TE VSM errors was performed to test the accuracy of the error estimation in Equation (3). Modeled data were obtained taking DOCMA FMs as ω(t) and reproducing jittered‐TE phase images by multiplying ω(t) by the corresponding TE: $\varphi_{odd/even}(t)=2\pi \omega_{odd/even}(t){\cdot TE}_{odd/even}$. This phase was used to calculate jittered‐TE FMs from adjacent time points. The difference between modeled jittered‐TE VSMs and DOCMA VSMs was calculated, and root‐mean‐square error maps were generated.

#### Experiment 3: Comparison of the Accuracy of the Static and Jittered‐TE Dynamic Distortion Correction in the Presence of Motion

The extent of head rotation was estimated using the SPM8 realignment tool 28. Static FMs were masked and smoothed, extrapolating signal beyond the brain boundary. VSMs derived from static FMs were applied to themselves, “forward warping” them to the EPI space. Jittered‐TE FMs were also smoothed but not masked (as they matched brain boundaries in the corresponding EPI volumes well) and converted to VSMs. In all cases, smoothing was applied using a discretized spline smoother [MATLAB function *smoothn.m* 29] with the smoothing parameter equal to 1. Combined magnitude EPI data were statically and dynamically corrected using the corresponding static or jittered‐TE VSMs. Because voxel shifts are generally non‐integer, linear interpolation in phase‐encode (PE) direction (MATLAB function *interp1.m*) was used to regrid the unwarped data to the original 128 × 128 matrix. Unwarped and original EPI data were motion corrected with respect to distortion‐free GE reference, and residual distortions were quantified by visual inspection in the MRIcro software.

#### Experiment 4: Evaluation of the Performance of Jittered‐TE DDC in a Block Design fMRI Task

Magnitude fluctuations arising from TE jittering were removed by calculating the mean intensity in odd and even volumes ( ${\bar{I}}_{odd}$, ${\bar{I}}_{even}$) and multiplying even volumes by the ratio ${\bar{I}}_{odd}/{\bar{I}}_{even}$. Standard EPI and intensity‐corrected jittered‐TE data were slice‐timing and motion corrected to the GE reference prior to general linear model analysis. Preprocessing and statistical analysis was performed with SPM8 28. Task‐related signal change was modeled with a box‐car function convolved with a canonical hemodynamic response function. Broad anatomical ROIs were manually defined around the hand and foot areas of the primary motor cortex. The mean suprathreshold *t* values in hand and foot ROIs (*P* < 0.05, family‐wise error corrected for multiple comparisons) were calculated for each subject. Wilcoxon sign‐rank paired tests with *P* < 0.05 were performed for the number of suprathreshold voxels and the mean suprathreshold *t* value in hand and foot areas (four tests) to assess possible differences in the BOLD sensitivity between standard and jittered‐TE EPI.

In order to assess potential difference in the localization of primary hand and foot regions with and without distortion correction, jittered‐TE EPI runs were additionally unwarped using the SDC or jittered‐TE DDC approach, as in experiment 3, prior to SPM8 preprocessing and general linear model analysis. Activation results were compared visually. Distortions in the proximity of the hand and foot area of the motor cortex were estimated from the original (noDC), SDC, and DDC magnitude data with respect to the distortion‐free GE reference. This analysis was performed in small cuboid ROIs, covering 8–25 mm in the readout direction and 16–33 mm in the PE direction and 2–4 slices. The matrix size in the PE direction was expanded by a factor of 20, allowing subvoxel shifts to be detected. Distorted and unwarped EPI magnitude values were iteratively shifted up or down along the PE direction in steps corresponding to 0.05 voxels in the original data. A Pearson correlation coefficient was calculated between noDC, SDC, or DDC EPI and a reference GE magnitude for each shift and for each PE line in the ROI. The extent of distortion was taken to be the value of shift corresponding to the highest correlation coefficient.

## RESULTS

### Experiment 1: Estimation of Respiration‐Induced Phase Fluctuations

The mean phase difference between respiration maxima and minima over subjects was 0.2 ± 0.1 rad in the most ventral slice. This decreased in dorsal direction, consistent with previous reports 8, 10, 11. The mean respiration frequency, estimated from images, was 0.237 ± 0.065 Hz, in agreement with the literature 11. If TR ≥ 2.1s (is above Nyquist rate), the maximum frequency change between adjacent time points due to respiration can thus reach δω = 0.2/(2π · 0.022) = 1.45 Hz. Corresponding jittered‐TE VSM errors can be estimated from Equation (3) using this value of δω and converting to voxel shifts according to Equation (2). Table 1 lists VSM errors expected with sequence parameters from experiments 2, 3, and 4. VSM errors decrease with increasing ΔTE, and are less than 0.3 voxels for ΔTE = 6 ms. Increasing ΔTE to 8 ms would reduce VSM errors by <1%. Note, however, that this calculation models respiration effects only.

**Table 1:** Voxel Shift Map Errors Estimated for Odd and Even Time Points and the Sequence Parameters Specified in experiment 2 and experiments 3 and 4

ΔTE (ms)	Estimated VSM Errors in Odd/Even Time Points (Voxels)			
	Parameters as in experiment 2, ${TE}_{odd/even}=\left[11,11+\Delta TE\right]$ ms		Parameters as in experiments 3 and 4, ${TE}_{odd/even}=\left[22‐\frac{\Delta TE}{2},22+\frac{\Delta TE}{2}\right]$ ms	
	Odd	Even	Odd	Even
0.8	0.46	0.43	1.79	1.73
2	0.20	0.17	0.74	0.67
4	0.12	0.09	0.38	0.32
6	0.09	0.06	0.27	0.20
8	0.07	0.04	0.21	0.14

### Experiment 2: Quantification of Respiration and Motion‐Induced Field Mapping Errors and Optimization of the Jittered‐TE Sequence

An example of modeled and experimental jittered‐TE VSM error maps and plots is shown in Figure 1. Data with ΔTE = 0.8 and ΔTE = 6.0 ms from a single representative subject performing no intentional motion were chosen. Modeling using a DOCMA FM to define the frequency offset allowed the spatial distribution and temporal fluctuations of jittered‐TE VSM errors to be quantified. For the scan with ΔTE = 0.8 ms, these were substantially larger than with ΔTE = 6.0 ms. The frequency change due to respiration (in no‐motion datasets) between adjacent time points reached up to 1 Hz, which is in agreement with the result in experiment 1 (1.45 Hz for the worst‐case scenario).

**Figure 2:**
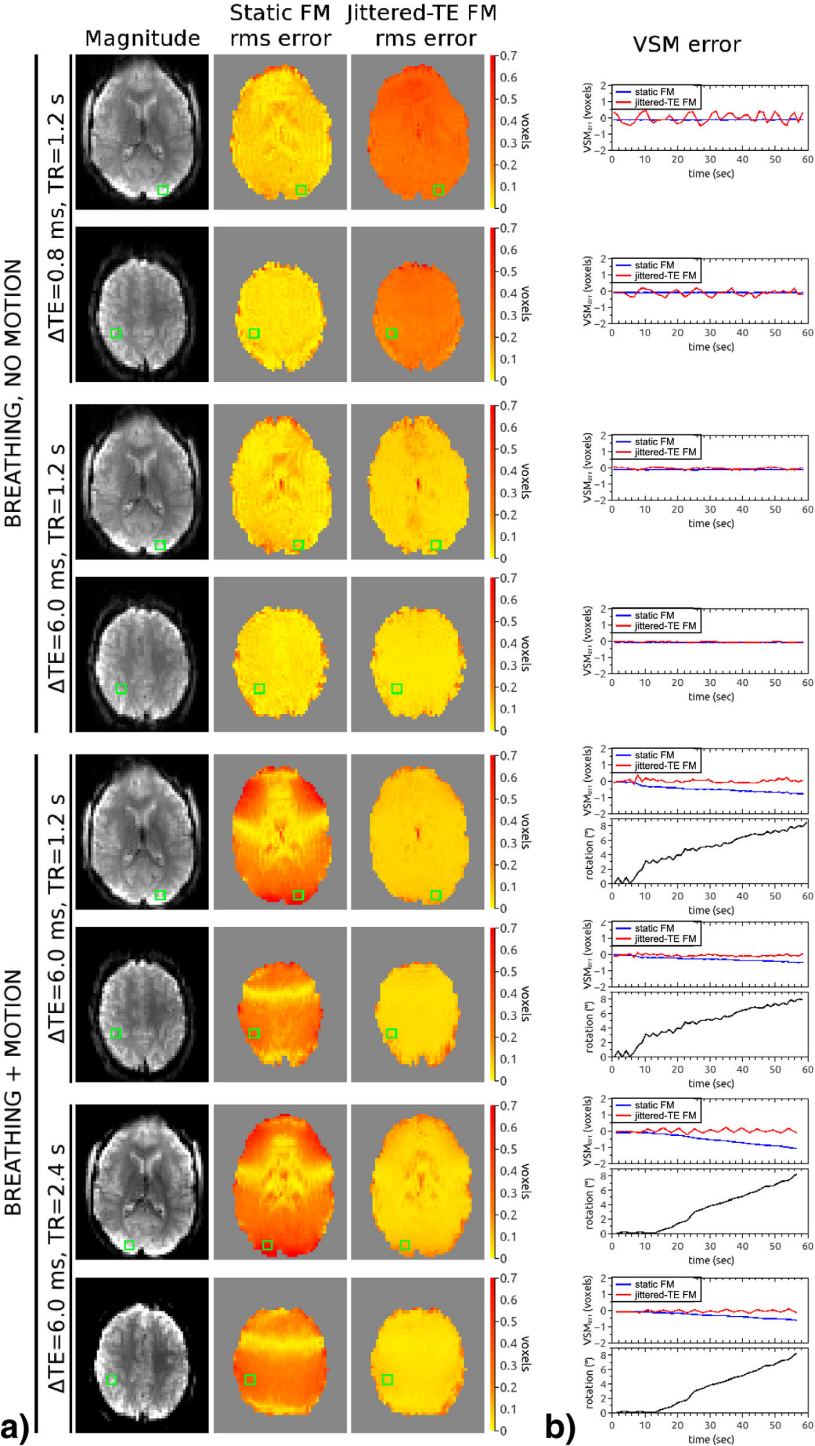
Quantification of static and jittered‐TE VSM errors in the breathing and breathing + motion conditions (ie, a slow nod). The values of ΔTE are 0.8 and 6.0 ms for the no motion and 6.0 ms for the motion condition. Two TRs were used in the motion condition: 1.2 s and 2.4 s. (**a**) Root‐mean‐square error maps. (**b**) Jittered‐TE and static VSM errors in selected ROIs (green squares from panel a) as a function of time. SPM8 rotation estimates (pitch + roll + yaw) are plotted in black for the breathing + motion condition.

Jittered‐TE and static VSM error maps were compared for both the “breathing, no motion” and “breathing + motion” conditions (Fig. 2). With a short ΔTE of 0.8 ms, respiration‐related errors were higher in jittered‐TE VSMs than in static VSMs (Fig. 2a, first and second row). The difference between DOCMA and jittered‐TE VSMs fluctuated between (−0.5, 0.5) voxels in the ROI in a ventral slice (Fig. 2b, first row) and between (0.2, −0.4) voxels in the ROI in a dorsal slice (Fig. 2b, second row). Jittered‐TE VSM errors were reduced with increasing ΔTE, reaching a level similar to that observed in static VSM for ΔTE = 6.0 ms (Fig. 2, third and fourth row). In the measurements with ΔTE = 6.0 ms and the slow nod, errors were much higher for static VSMs (Fig. 2, fifth through eighth rows). The plots illustrate how the static VSMs diverge from DOCMA VSMs with head movement while jittered‐TE VSMs remain accurate. In the ROI from a ventral slice, static VSM errors reached a value of 0.8 voxels for the data with TR = 1200 ms and cumulative rotation (ie, pitch + roll + yaw) up to 8.5° (Fig. 2b, fifth row) and 1.0 voxels for the data with TR = 2400 ms and rotation up to 8.2° (Fig. 2b, seventh row). The errors in the jittered‐TE VSMs increased slightly with TR, reaching a maximum of about 0.2 voxels for TR = 2400 ms when respiration and motion was present (compare Fig. 2, rows 5 and 6 with Fig. 2, rows 7 and 8). This analysis, based on low‐resolution (3.3 × 3.3 × 4.4 mm^3^) data, together with the estimation of the respiration errors for a sequence with higher resolution (1.64 × 1.64 × 2.0 mm^3^) from experiment 1, was the basis for the choice of ΔTE = 6.0 ms for experiments 3 and 4.

### Experiment 3: Comparison of the Accuracy of the Static and Jittered‐TE Dynamic Distortion Correction in the Presence of Motion

**Figure 3:**
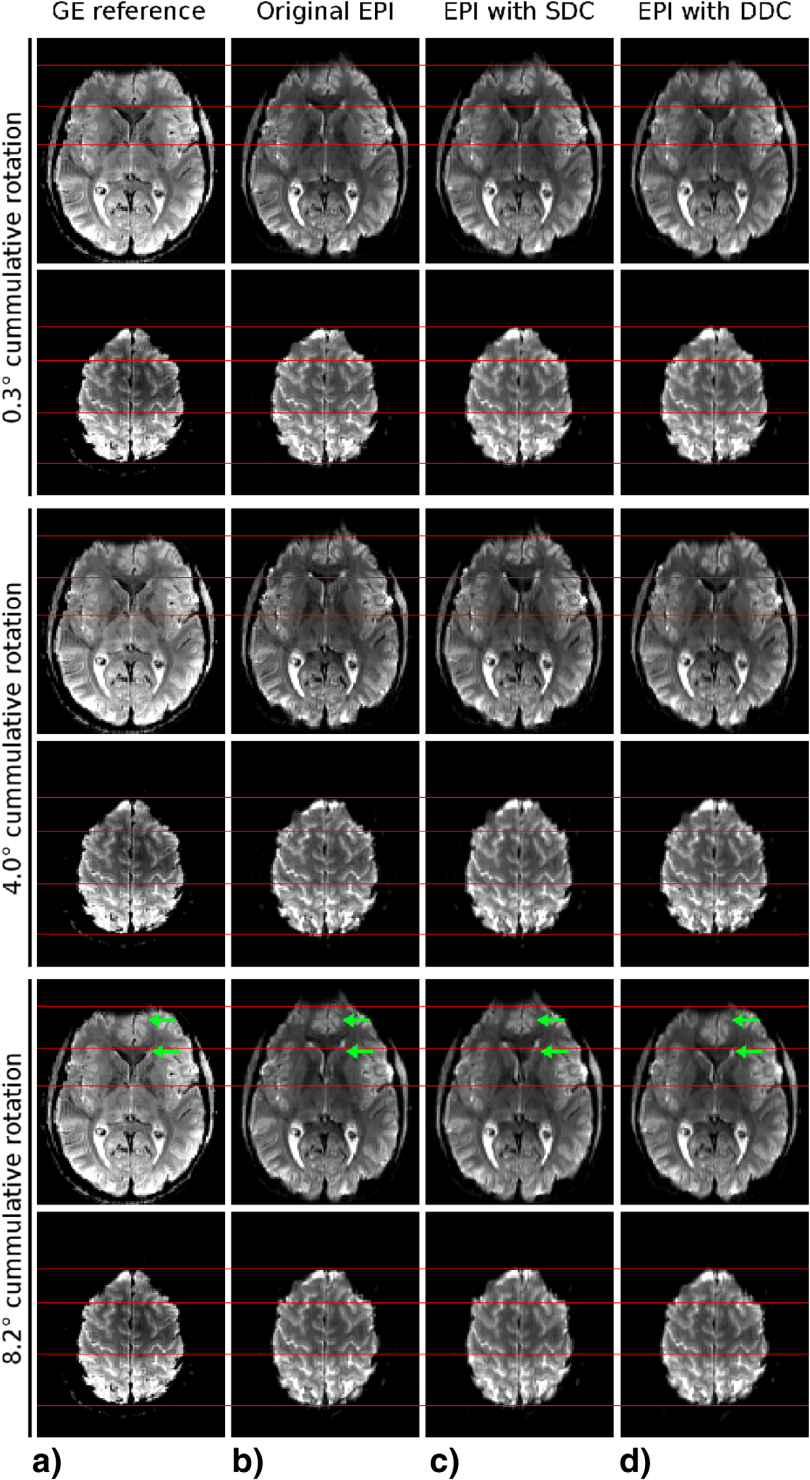
(**a**) A distortion‐free GE reference for volunteer V1 with cumulative head rotation up to 8.2° performed during EPI acquisition compared with (**b**) the degree of distortion in raw EPI, (**c**) the accuracy of SDC, and (**d**) the accuracy of jittered‐TE DDC. Red lines highlight structures of interest (eg, brain boundaries, central sulcus). Distortions in SDC data reached up to 8.2 mm at the brain boundary and close to sinuses (green arrows, fifth row). Unwarping with the jittered‐TE method left no residual distortions.

Higher resolution data (1.64 × 1.64 × 2.0 mm^3^) with SDC and jittered‐TE DDC in the presence of motion are presented for one subject in Figure 3 and two other subjects in Supporting Figures S1 and S2. Two slices are shown at three time points; those with the smallest, intermediate, and largest cumulative rotations with respect to the GE reference. There were unwarping errors in all SDC images (column c) in each figure, which increased with head rotation. SDC leads to blurring and incorrect correction close the ventricles, even for the smallest rotation (0.3°) (Fig. 3c, top row). For the maximum head rotation, deformations in SDC data reached 8.2 mm for the first two volunteers (Fig. 3 and Supporting Fig. S1, ventral slice, green arrows) and 6.6 mm for the third volunteer (Supporting Fig. S2, green arrows). Unwarping errors of about 1.6 mm were visible close to the inverted omega of the central sulcus in Supporting Figure S2 (dorsal slice, green arrows). No residual distortions were apparent after jittered‐TE DDC.

### Experiment 4: Evaluation of the Performance of Jittered‐TE DDC in a Block Design fMRI Task

**Figure 4:**
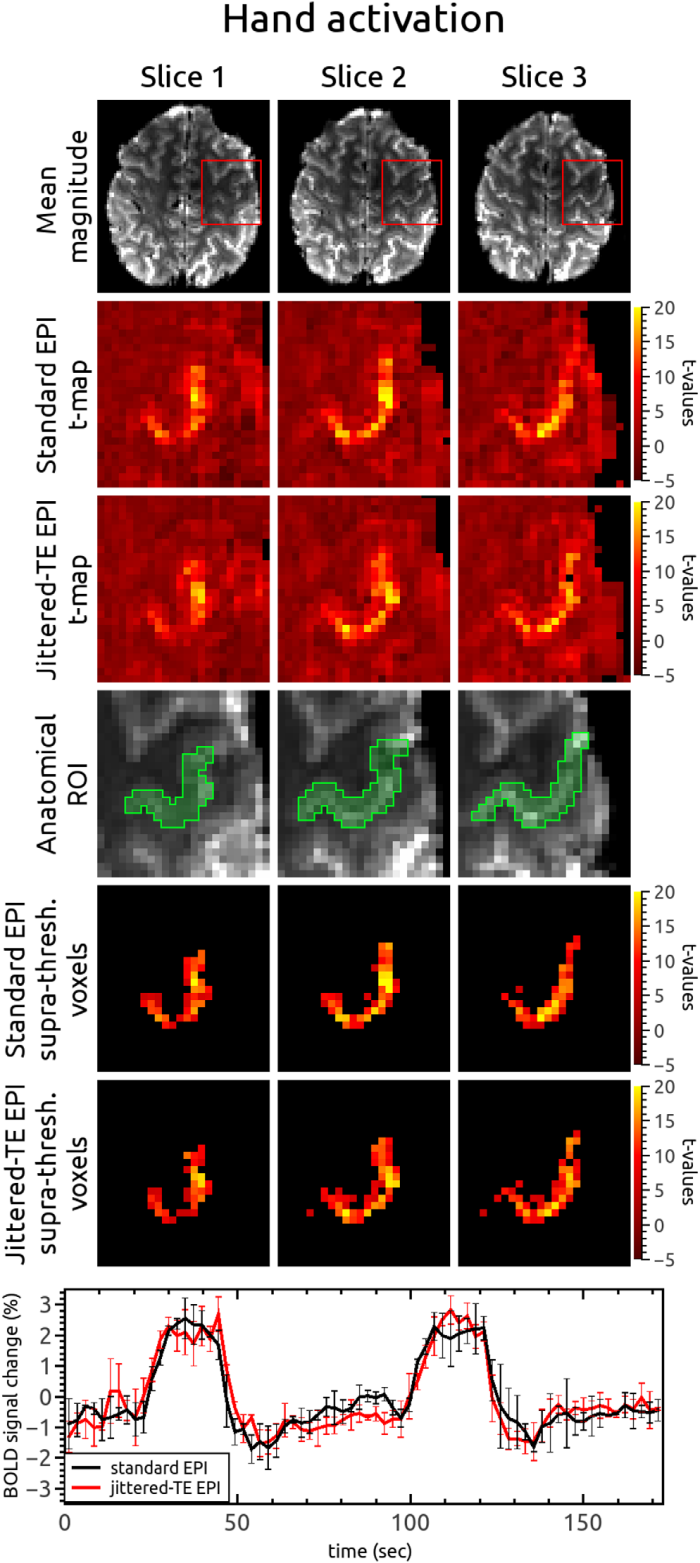
Comparison of hand activation maps from volunteer V1 derived from standard (second row) and jittered‐TE (third row) EPI runs without distortion correction. The fourth row shows a manually defined anatomical ROI in the hand region of the primary motor cortex. Suprathreshold voxels from *t* maps in the anatomical ROI are shown in the fifth row for standard and in the sixth row for jittered‐TE EPI. In the bottom row, the mean BOLD signal changes in suprathreshold voxels is plotted for standard EPI (black) and jittered‐TE EPI (red), showing very similar behavior.

This experiment was performed to assess whether the BOLD sensitivity of the jittered‐TE sequences was compromised, compared with standard EPI, by changes to the echo time. No distortion correction was applied to either the standard or jittered‐TE data. Sample hand activation maps from the two sequences are shown in Figure 4. The distribution of activated voxels in the two sequences was similar (Fig. 4, second and third row). Voxels with *P* < 0.05 from standard and jittered‐TE *t* maps in the anatomical ROI are shown in the fifth and sixth rows of Figure 4. The mean BOLD signal changes in suprathreshold voxels over three runs are presented at the bottom of Figure 4, and show that 1) there are no residual fluctuations in the magnitude between odd and even time points following intensity correction (red line) and 2) the two methods have similar BOLD sensitivity. Similar results were obtained for the activated foot area (Supporting Fig. S3). A summary of hand and foot activation results for all volunteers is presented in Table 2. Wilcoxon signed‐rank paired tests showed that there was no significant difference between the standard and jittered‐TE sequence in the number of suprathreshold voxels or the mean suprathreshold *t* value (see Table 2, mean over volunteers).

**Table 2:** Quantification of Mean t Values and the Number of Suprathresholded (P < 0.05) Voxels in Hand and Foot Functional ROIs for fMRI Performed with Standard and jittered‐TE EPI

Volunteer	Hand ROI				Foot ROI			
	Mean *t* Value		Number of Voxels		Mean *t* Value		Number of Voxels	
	Standard	jittered‐TE	Standard	jittered‐TE	Standard	jittered‐TE	Standard	jittered‐TE
V1	10.8	9.4	130	103	7.9	7.5	62	42
V2	11.0	11.5	190	200	8.0	7.8	95	93
V3	11.3	11.6	201	247	9.0	9.6	27	63
V4	10.8	10.0	248	237	9.5	8.4	106	106
V5	11.6	10.7	266	241	8.1	8.4	155	150
V6	11.1	10.3	247	250	8.4	8.1	138	132
Mean over volunteers	11.1	10.6	214	213	8.5	8.3	97	98
Paired test *P* values^a^	0.12		0.75		0.46		0.50	

Four tests were performed with standard‐jittered‐TE pairs of values; the difference between either the mean *t* values or the number of suprathresholded voxels was tested.

a *P* values obtained from Wilcoxon signed‐rank paired tests at the *P* < 0.05 significance level.

**Figure 5:**
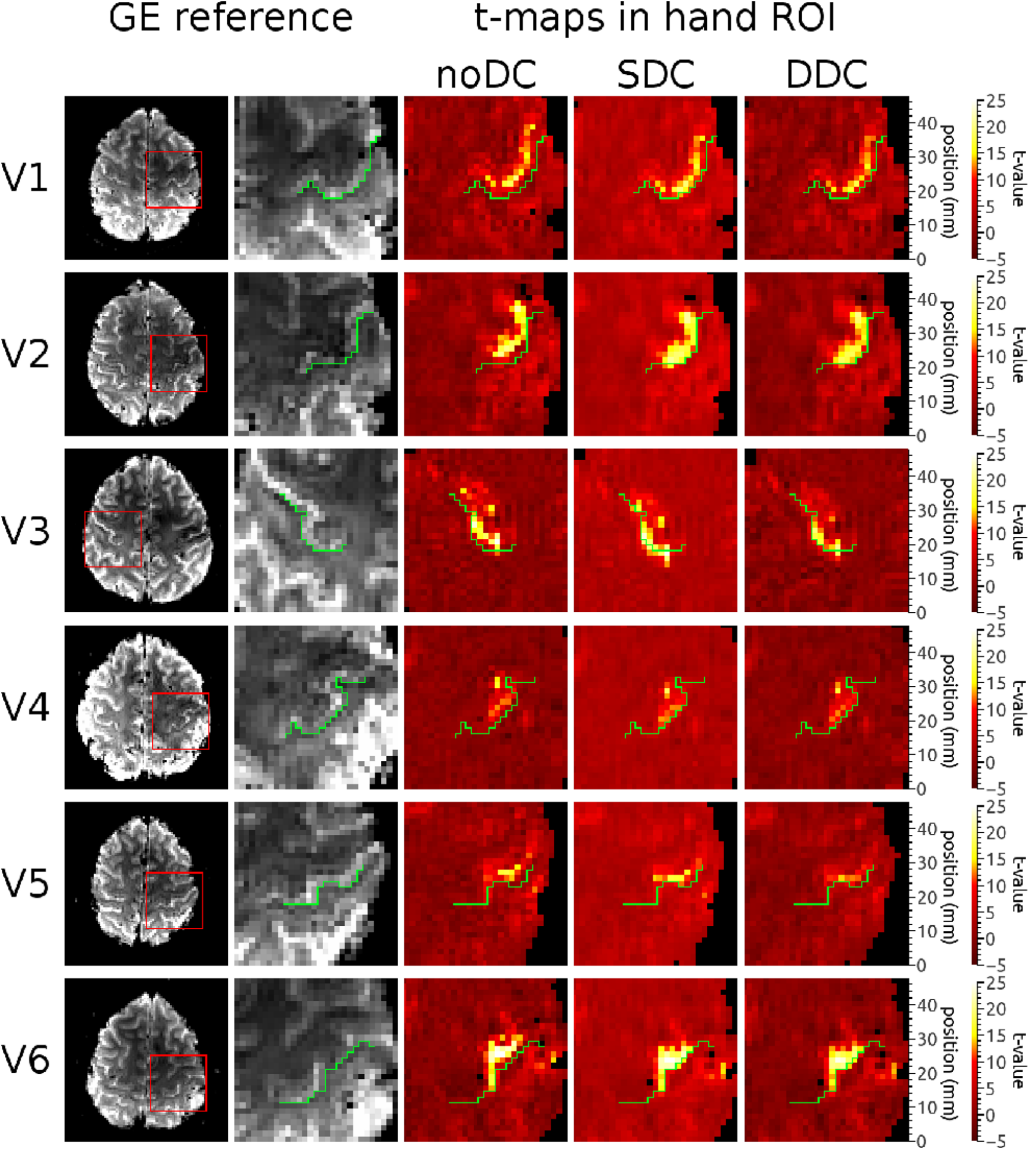
Comparison between activation maps in a hand ROI after no (noDC), static (SDC), or jittered‐TE dynamic (DDC) distortion correction for all volunteers participating in the motor task fMRI experiment. The hand ROI is marked by the red rectangle in the reference GE image. The green line in the enlarged GE ROI marks the posterior border of the central sulcus. The same line was marked on all activation maps. After DDC, activation for all volunteers was located on the anterior wall of the central sulcus and followed its curvature, as is expected for the hand knob area. After SDC, the activation was located on the anterior wall of the central sulcus in five of six volunteers (all except V3).

Hand activation results after noDC, SDC, or jittered‐TE DDC are compared in Figure 5. The green line is the outline of the posterior boundary of the central sulcus, which was traced on the distortion‐free GE reference (column 1). For each volunteer (V1‐V6), the activation results originate from the same three jittered‐TE EPI runs after different unwarping procedures. The activated region in the noDC *t* map was located anterior to the central sulcus for all the volunteers, with a clearly visible gap for V1, V2, V3, and V6 (of about 1–2 voxels). In V3, the SDC shifted a part of the activated region posterior to the central sulcus. For all subjects after DDC and for five subjects (all except V3) after SDC, activation was located on the anterior wall of the central sulcus, following its shape, as expected for the hand knob area 30. The *t* values in the SDC results were slightly higher, especially for the voxels outside the primary hand region (background), and more blurred than in noDC and DDC results.

Distortion estimates in the proximity of activated hand and foot areas are summarized in Table 3. For all volunteers, the mean distortion in the noDC data was ≥1.6 mm, reaching a maximum of 3.9 mm for a hand (V1) and 6.3 mm for a foot area (V5). The distortions were partially reduced by the SDC. The DDC led in some cases to similar results (eg, V1 and V2 foot ROI), but in most cases to a greater reduction of distortions. For V3, in which the hand activation after SDC was shifted posterior to the central sulcus (Fig. 5), the mean distortion in the hand ROI after SDC was equal to 1 mm and reached a maximum of 1.7 mm. The distortions in V3 were reduced by DDC to 0.1 mm on average with the maximum being 0.4 mm. In the foot ROI, the largest discrepancy between the SDC and the DDC was for V5, where the maximum distortions in noDC data of 6.3 mm was reduced to 1.2 mm by the SDC to 0.2 mm by the DDC. Visual inspection showed the effectiveness of SDC to be further reduced close to the sinuses, as in experiment 3. For V3 and V5, those distortions in SDC data reached up to 6.6 mm (4 voxels). No residual distortions were apparent in this region in DDC results.

**Table 3:** Comparison of Relative Position of Structure of Interest Between Not Corrected (noDC), Statically (SDC), and Dynamically (DDC) Unwarped EPI Data and Distortion‐Free GE Data

ROI and Volunteer Number	No. of voxels in ROI	Distortions in the Proximity of an Activated Area (in mm)								
		noDC			SDC			DDC		
		Mean	SD	Maximum	Mean	SD	Maximum	Mean	SD	Maximum
Hand ROI										
V1	924	2.5	0.4	3.9	0.1	0.1	0.7	0.1	0.1	0.6
V2	720	2.5	0.4	3.3	0.2	0.2	0.7	0.1	0.3	0.7
V3	1056	1.9	0.6	3.6	1.0	0.3	1.7	0.1	0.1	0.4
V4	1224	2.1	0.4	2.9	0.5	0.3	1.3	0.2	0.2	0.8
V5	780	1.6	0.5	3.0	0.5	0.5	1.6	0.2	0.2	0.7
V6	306	2.2	0.3	2.7	0.3	0.2	0.7	0.2	0.2	0.7
Foot ROI										
V1	408	2.7	0.8	3.5	0.2	0.2	0.7	0.2	0.2	0.7
V2	135	3.5	0.4	4.4	0.3	0.3	0.8	0.3	0.3	0.8
V3	504	1.8	0.3	2.4	0.3	0.2	0.6	0.1	0.0	0.2
V4	756	2.0	0.7	3.1	0.5	0.4	1.2	0.3	0.3	0.9
V5	399	1.5	1.2	6.3	0.5	0.5	1.2	0.1	0.0	0.2
V6	399	2.2	0.3	2.7	0.2	0.2	0.7	0.1	0.0	0.2

Abbreviation: SD, standard deviation.

## DISCUSSION

We have presented a method for dynamic distortion correction based on single‐echo EPI. A jittering of the TE between adjacent EPI volumes allows FMs to be calculated between consecutive time points. This approach has been tested at 7T, where prominent static and dynamic distortions are well documented. We have shown that those distortions are accurately corrected with jittered‐TE DDC if appropriate sequence parameters are used, and that an SDC fails to fully correct or even increases distortions if the head is rotated by a few degrees during the EPI time series. In the presence of task‐related motion, distortion was reduced more effectively by jittered‐TE DDC than the SDC approach. SDC was also found to blur activation, an effect not observed with the jittered‐TE DDC. The BOLD sensitivity of a sequence with jittered TE_odd,even_ = [19,25] ms was comparable to that of a standard EPI sequence with TE = 22 ms.

To our knowledge, this is the first study at 7T to investigate the performance of a DDC method based on single‐echo EPI. Other DDC approaches have been suggested, based on periodic variation in phase blips 31, echo time, or gradient reversal 32, although neither study presented results for the dynamic variant. Prior work using single‐echo EPI‐based DDC has also assessed performance at 3T or lower field strengths, has required a reference scan for a complete distortion correction, has been founded on the assumption that the $\varphi_{o}$ is constant over the fMRI measurement time 17, 18, 19, 20, and has used volume receive coils 18, 20.

A separate scan is required for the SDC method 33, 34 and for dynamic methods based on the temporal stability of $\varphi_{o}$. As well as increasing measurement time, the need for a separate scan makes the experiment more prone to inconsistent geometry or shim between the reference scan and EPI to be corrected, especially when a large number of runs is performed. Jittered‐TE DDC requires no reference scan, obtaining all the necessary information directly from the EPI in each fMRI run.

Jittered‐TE FMs do not accurately represent the ΔB_0_ at two time points if there is a substantial change in the field between the two (eg, due to motion or respiration). Our estimates of the average respiration frequency (0.237 ± 0.065 Hz) and the maximum respiration‐induced field changes in healthy subjects at 7T (1.45 Hz) are in good agreement with the literature 8, 10, 11. We have shown that jittered‐TE FM errors due to respiration can be estimated analytically for any sequence parameters using the maximum value of respiration‐induced field changes. With the parameters chosen for the motor task fMRI, those errors were <0.5 mm. This analysis allows errors at other field strengths to be predicted. At 3T, for instance, we would expect the maximum respiration‐induced field changes to be 1.45 · 3/7 = 0.62 Hz. With the same TR, ΔTE, RBW, and voxel dimensions as were used in this study, and TEs around a value typically used at 3 T (ie, TE_odd,even_ = [32,38] ms), errors up to about 0.3 mm would be expected. At 9.4 T, with field changes of 1.45 · 9.4/7 = 1.95 Hz and TE_odd,even_ = [15,21] ms, we would expect errors up to 0.5 mm, similar to those observed at 7T.

In this study, we demonstrated that jittered‐TE FM errors can be reduced to a low level by an appropriate selection of TE, ΔTE, and TR. TR is usually constrained by the desired coverage and TE by BOLD sensitivity. There is generally more freedom in the selection of ΔTE. Increasing ΔTE reduces errors in jittered‐TE FMs but risks reducing BOLD contrast and increasing TR. A compromise was made here with ΔTE = 6 ms, which yielded residual erroneous shifts of a small fraction of a voxel while requiring changes to TE, which in turn had a modest influence on TR and no observable effect on BOLD sensitivity [given the broad maximum in the BOLD sensitivity versus TE curve 35].

Using DOCMA as a reference in experiment 2, we showed that the optimized jittered‐TE FMs depicted ΔB_0_ inhomogeneities with high accuracy. Because the jittered‐TE DDC is not subject to the spatio‐temporal limitations of the DOCMA approach, we also performed measurements with higher spatial resolution in experiments 3 and 4 and used distortion‐free GE scans as a reference. Experiment 3 demonstrated that jittered‐TE DDC accurately unwarps images when large, slow motion is present, whereas the SDC can increase geometric distortions, mainly close to brain boundaries and sinuses. If large and abrupt movement was present in the middle of EPI time series (eg, between volume *n* and *n + 1*), the errors in the corresponding jittered‐TE FMs can be significant. This could be remedied by identifying the volumes affected (eg, using motion estimates) and substituting the affected FM by the closest unaffected FMs or by an interpolation between them. The Pearson correlation analysis in experiment 4 showed residual distortions in SDC reaching 1.7 mm in the hand and 1.2 mm in the foot ROI. Residual distortions in DDC, due to motion and breathing, were generally smaller than in the SDC case (maximum always below 1 mm). Distortions of up to 6.6 mm were recognized in the SDC results close to the sinuses, where the DDC left no visible distortion. This is of potential importance in fMRI studies of emotions that elicit prefrontal cortex and anterior cingulate cortex activation 36, 37. The differences between the uncorrected and the statically and dynamically unwarped data could be much larger in patients and children, who usually move more 38, 39.

FMs estimated in the EPI space (as in jittered‐TE DDC) do not allow signal pileup, which can occur due to nonlinear distortions to be resolved. Using a posterior−anterior phase encoding direction ensures that signal pile‐ups are negligible. In the motor cortex, signal is in fact stretched rather than piled‐up 40.

Any distortion correction that uses VSM introduces blurring, because of the interpolation used to regrid the unwarped data to the original matrix. This was more apparent in SDC than DDC, however, especially in experiment 3 and the activation maps from experiment 4.

It is possible to calculate a VSM between two single‐echo EPI acquisitions without changing the TE by “jittering” the gradient moment of the phase prewinder in adjacent volumes between two values that differ by an integer number of phase blips 33. This is subject to the same VSM errors as jittered‐TE method from phase changes between adjacent time points. A single PE blip shifts the data acquisition window in k‐space by one PE line, which corresponds to the time of a single echo spacing (here 0.75 ms) and would produce similar errors to our measurements with ΔTE = 0.8 ms. Increasing the prewinder jitter to multiple PE blips in order to reduce VSM errors could lead to type 2 signal loss 41, especially if partial Fourier and acceleration were used, because of the low number of acquired k‐space lines.

The jittered‐TE approach is especially suited for short TR applications, as reducing TR generally reduces jittered‐TE FM errors. Additional benefits over those shown here are therefore expected when combining the jittered‐TE DDC with simultaneous multislice/multi‐band EPI 42, 43, 44 or highly accelerated 3D EPI 45.

## CONCLUSIONS

Jittered‐TE DDC is a dynamic distortion correction approach based on single‐echo EPI. It requires the echo time to be alternated between odd and even time points in EPI, which allows the calculation of FMs dynamically, directly from the fMRI data. There is no need for a reference scan, and with appropriate TEs and TR the approach yields accurate FMs even in the presence of motion. Spatio‐temporal resolution is not compromised, and there was no observable reduction in BOLD sensitivity compared to conventional EPI in the experiment design used here. The method can be applied in a wide range of fMRI experiments, especially those in which substantial motion is expected.

## Supporting information

Supporting Information
**Supporting Figure S1.** (**a**) A distortion‐free GE reference for volunteer V2 with cumulative head rotation up to 7.8^°^ performed during EPI acquisition compared with (**b**) the degree of distortion in raw EPI, (**c**) the accuracy of SDC, and (**d**) the accuracy of jittered‐TE DDC. Red lines highlight structures of interest (eg, brain boundaries, central sulcus). Distortions in SDC data reached up to 8.2 mm at the brain boundary (green arrows, fifth row). Unwarping with the jittered‐TE method left no residual distortions.
**Supporting Figure S2.** (**a**) A distortion‐free GE reference for volunteer V4 with the cumulative head rotation up to 6.9^°^ performed during EPI acquisition compared with (**b**) the degree of distortion in raw EPI, (**c**) the accuracy of SDC and (**d**) the accuracy of jittered‐TE DDC. Red lines highlight structures of interest (eg, brain boundaries, central sulcus). Distortions in SDC data reached up to 6.6 mm (green arrows, fifth row) and showed residual distortions of approximately 1.6 mm around the central sulcus (green arrows, sixth row). Unwarping with the jittered‐TE method left no residual distortions.
**Supporting Figure S3.** Comparison of foot activation maps from volunteer V1 derived from standard (second row) and jittered‐TE (third row) EPI runs without distortion correction. The fourth row shows a manually defined anatomical ROI in the foot region of the primary motor cortex. Suprathreshold voxels from *t* maps in the anatomical ROI are shown in the fifth row for standard EPI and in the sixth row for jittered‐TE EPI. In the bottom row, mean BOLD signal change in suprathreshold voxels is plotted for standard (black) and jittered‐TE EPI (red), showing very similar behavior.

## References

[1] Turner R , Jezzard P , Wen H , Kwong KK , Le Bihan D , Zeffiro T , Balaban RS. Functional mapping of the human visual cortex at 4 and 1.5 tesla using deoxygenation contrast EPI. Magn Reson Med 1993;29:277–279. 8429797 10.1002/mrm.1910290221

[2] Gati JS , Menon RS , UĂurbil K , Rutt BK. Experimental determination of the BOLD field strength dependence in vessels and tissue. Magn Reson Med 1997;38:296–302. 9256111 10.1002/mrm.1910380220

[3] Krüger G , Kastrup A , Glover GH. Neuroimaging at 1.5 T and 3.0 T: comparison of oxygenation‐sensitive magnetic resonance imaging. Magn Reson Med 2001;45:595–604. 11283987 10.1002/mrm.1081

[4] van der Zwaag W , Francis S , Head K , Peters A , Gowland P , Morris P , Bowtell R. fMRI at 1.5, 3 and 7 T: characterising BOLD signal changes. NeuroImage 2009;47:1425–1434. 19446641 10.1016/j.neuroimage.2009.05.015

[5] Jezzard P. Correction of geometric distortion in fMRI data. NeuroImage 2012;62:648–651. 21945795 10.1016/j.neuroimage.2011.09.010

[6] Hutton C , Bork A , Josephs O , Deichmann R , Ashburner J , Turner R. Image distortion correction in fMRI: a quantitative evaluation. Neuroimage 2002;16:217–240. 11969330 10.1006/nimg.2001.1054

[7] Hutton C , Andersson J , Deichmann R , Weiskopf N. Phase informed model for motion and susceptibility. Hum Brain Mapp 2013;34:3086–3100. 22736546 10.1002/hbm.22126 PMC6870252

[8] Van de Moortele P‐F , Pfeuffer J , Glover GH , Ugurbil K , Hu X. Respiration‐induced B0 fluctuations and their spatial distribution in the human brain at 7 Tesla. Magn Reson Med 2002;47:888–895. 11979567 10.1002/mrm.10145

[9] Barry RL , Menon RS. Modeling and suppression of respiration‐related physiological noise in echo‐planar functional magnetic resonance imaging using global and one‐dimensional navigator echo correction. Magn Reson Med 2005;54:411–418. 16032665 10.1002/mrm.20591

[10] Zeller M , Kraus P , Müller A , Bley TA , Köstler H. Respiration impacts phase difference‐based field maps in echo planar imaging. Magn Reson Med 2013;72:446–451. 24018714 10.1002/mrm.24938

[11] Zahneisen B , Assländer J , LeVan P , Hugger T , Reisert M , Ernst T , Hennig J. Quantification and correction of respiration induced dynamic field map changes in fMRI using 3D single shot techniques. Magn Reson Med 2014;71:1093–1102. 23716298 10.1002/mrm.24771

[12] Jezzard P , Balaban RS. Correction for geometric distortion in echo planar images from B0 field variations. Magn Reson Med 1995;34:65–73. 7674900 10.1002/mrm.1910340111

[13] Schmithorst VJ , Dardzinski BJ , Holland SK. Simultaneous correction of ghost and geometric distortion artifacts in EPI using a multiecho reference scan. IEEE Trans Med Imaging 2001;20:535–539. 11437113 10.1109/42.929619 PMC1357361

[14] Zeller M , Müller A , Hahn D , Köstler H. Phase‐labeled reference EPI for frequency‐segmented inhomogeneity corrections (PREFICS). Magn Reson Med 2014;71:1117–1122. 23554070 10.1002/mrm.24737

[15] Poser BA , Versluis MJ , Hoogduin JM , Norris DG. BOLD contrast sensitivity enhancement and artifact reduction with multiecho EPI: parallel‐acquired inhomogeneity‐desensitized fMRI. Magn Reson Med 2006;55:1227–1235. 16680688 10.1002/mrm.20900

[16] Visser E , Poser BA , Barth M , Zwiers MP. Reference‐free unwarping of EPI data using dynamic off‐resonance correction with multiecho acquisition (DOCMA). Magn Reson Med 2012;68:1247–1254. 22851507 10.1002/mrm.24119

[17] Marques JP , Bowtell R. Evaluation of a New Method to Correct the Effects of Motion‐Induced B0‐field Variation During fMRI. In Proceedings of the 13th Annual Meeting of ISMRM, Miami Beach, Florida, USA, 2005. Abstract 510.

[18] Lamberton F , Delcroix N , Grenier D , Mazoyer B , Joliot M. A new EPI‐based dynamic field mapping method: application to retrospective geometrical distortion corrections. J Magn Reson Imaging 2007;26:747–755. 17729370 10.1002/jmri.21039

[19] Hahn AD , Nencka AS , Rowe DB. Improving robustness and reliability of phase‐sensitive fMRI analysis using temporal off‐resonance alignment of single‐echo timeseries (TOAST). NeuroImage 2009;44:742–752. 18992826 10.1016/j.neuroimage.2008.10.001 PMC2884970

[20] Ooi MB , Muraskin J , Zou X , Thomas WJ , Krueger S , Aksoy M , Bammer R , Brown TR. Combined prospective and retrospective correction to reduce motion‐induced image misalignment and geometric distortions in EPI. Magn Reson Med 2012;69:803–811. 22499027 10.1002/mrm.24285 PMC3402592

[21] Robinson S , Grabner G , Witoszynskyj S , Trattnig S. Combining phase images from multi‐channel RF coils using 3D phase offset maps derived from a dual‐echo scan. Magn Reson Med 2011;65:1638–1648. 21254207 10.1002/mrm.22753

[22] Reeder SB , Atalar E , Faranesh AZ , McVeigh ER. Referenceless interleaved echo‐planar imaging. Magn Reson Med 1999;41:87–94. 10025615 10.1002/(sici)1522-2594(199901)41:1<87::aid-mrm13>3.0.co;2-x PMC2396321

[23] Beisteiner R , Robinson S , Wurnig M , et al. Clinical fMRI: evidence for a 7 T benefit over 3 T. NeuroImage 2011;57:1015–1021. 21620980 10.1016/j.neuroimage.2011.05.010 PMC3134943

[24] Yang Z , Huang Z , Gonzalez‐Castillo J , Dai R , Northoff G , Bandettini P. Using fMRI to decode true thoughts independent of intention to conceal. NeuroImage 2014;99:80–92. 24844742 10.1016/j.neuroimage.2014.05.034 PMC4179453

[25] Robinson S , Jovicich J. B0 mapping with multi‐channel RF coils at high field. Magn Reson Med 2011;66:976–988. 21608027 10.1002/mrm.22879

[26] Bernstein MA , Grgic M , Brosnan TJ , Pelc NJ. Reconstructions of phase contrast, phased array multicoil data. Magn Reson Med 1994;32:330–334. 7984065 10.1002/mrm.1910320308

[27] Jenkinson M. Fast, automated, N‐dimensional phase‐unwrapping algorithm. Magn Reson Med 2003;49:193–197. 12509838 10.1002/mrm.10354

[28] Friston KJ , Holmes AP , Worsley KJ , Poline J‐P , Frith CD , Frackowiak RSJ. Statistical parametric maps in functional imaging: a general linear approach. Hum Brain Mapp 1994;2:189–210.

[29] Garcia D. Robust smoothing of gridded data in one and higher dimensions with missing values. Comput Stat Data Anal 2010;54:1167–1178. 24795488 10.1016/j.csda.2009.09.020 PMC4008475

[30] White LE , Andrews TJ , Hulette C , Richards A , Groelle M , Paydarfar J , Purves D. Structure of the human sensorimotor system. I: morphology and cytoarchitecture of the central sulcus. Cereb Cortex 1997;7:18–30. 9023429 10.1093/cercor/7.1.18

[31] Xiang Q‐S , Ye FQ. Correction for geometric distortion and N/2 ghosting in EPI by phase labeling for additional coordinate encoding (PLACE). Magn Reson Med 2007;57:731–741. 17390358 10.1002/mrm.21187

[32] Pfeuffer J , Vogler M , inventors; Siemens Aktiengesellschaft, assignee. Method and apparatus for dynamic distortion correction in EPI measurements in medical magnetic resonance imaging. US patent US8040133 B2. October 18, 2011.

[33] Zeng H , Constable RT. Image distortion correction in EPI: comparison of field mapping with point spread function mapping. Magn Reson Med 2002;48:137–146. 12111941 10.1002/mrm.10200

[34] Zaitsev M , Hennig J , Speck O. Point spread function mapping with parallel imaging techniques and high acceleration factors: fast, robust, and flexible method for echo‐planar imaging distortion correction. Magn Reson Med 2004;52:1156–1166. 15508146 10.1002/mrm.20261

[35] Poser BA , Norris DG. Investigating the benefits of multi‐echo EPI for fMRI at 7 T. NeuroImage 2009;45:1162–1172. 19349231 10.1016/j.neuroimage.2009.01.007

[36] Berthoz S , Artiges E , Van de Moortele P‐F , Poline J‐B , Rouquette S , Consoli SM , Martinot J‐L. Effect of impaired recognition and expression of emotions on frontocingulate cortices: an fMRI study of men with alexithymia. Am J Psychiatry 2002;159:961–967. 12042184 10.1176/appi.ajp.159.6.961

[37] Mak AKY , Hu Z , Zhang JX , Xiao Z , Lee TMC. Neural correlates of regulation of positive and negative emotions: an fMRI study. Neurosci Lett 2009;457:101–106. 19429172 10.1016/j.neulet.2009.03.094

[38] Lemieux L , Salek‐Haddadi A , Lund TE , Laufs H , Carmichael D. Modelling large motion events in fMRI studies of patients with epilepsy. Magn Reson Imaging 2007;25:894–901. 17490845 10.1016/j.mri.2007.03.009

[39] Yuan W , Altaye M , Ret J , Schmithorst V , Byars AW , Plante E , Holland SK. Quantification of head motion in children during various fMRI language tasks. Hum Brain Mapp 2009;30:1481–1489. 18636549 10.1002/hbm.20616 PMC2763570

[40] De Panfilis C , Schwarzbauer C. Positive or negative blips? The effect of phase encoding scheme on susceptibility‐induced signal losses in EPI. NeuroImage 2005;25:112–121. 15734348 10.1016/j.neuroimage.2004.11.014

[41] Deichmann R , Josephs O , Hutton C , Corfield DR , Turner R. Compensation of susceptibility‐induced BOLD sensitivity losses in echo‐planar fMRI imaging. NeuroImage 2002;15:120–135. 11771980 10.1006/nimg.2001.0985

[42] Moeller S , Yacoub E , Olman CA , Auerbach E , Strupp J , Harel N , Uğurbil K. Multiband multislice GE‐EPI at 7 Tesla, with 16‐fold acceleration using partial parallel imaging with application to high spatial and temporal whole‐brain fMRI. Magn Reson Med 2010;63:1144–1153. 20432285 10.1002/mrm.22361 PMC2906244

[43] Feinberg DA , Moeller S , Smith SM , Auerbach E , Ramanna S , Glasser MF , Miller KL , Ugurbil K , Yacoub E. Multiplexed echo planar imaging for sub‐second whole brain FMRI and fast diffusion imaging. PLoS One 2010;5:e15710. 21187930 10.1371/journal.pone.0015710 PMC3004955

[44] Setsompop K , Gagoski BA , Polimeni JR , Witzel T , Wedeen VJ , Wald LL. Blipped‐controlled aliasing in parallel imaging for simultaneous multislice echo planar imaging with reduced g‐factor penalty. Magn Reson Med 2012;67:1210–1224. 21858868 10.1002/mrm.23097 PMC3323676

[45] Poser BA , Koopmans PJ , Witzel T , Wald LL , Barth M. Three dimensional echo‐planar imaging at 7 Tesla. NeuroImage 2010;51:261–266. 20139009 10.1016/j.neuroimage.2010.01.108 PMC2853246

