# Supplementary material for: Correcting dynamic distortions in 7T echo planar imaging using a jittered echo time sequence
Source: Magn Reson Med. 2015 Nov 19;76(5):1388–99. doi: 10.1002/mrm.26018 (PMC5082535; doi:10.1002/mrm.26018)
Supplement: Supplementary file 1 — Supporting Information Supporting Figure S1. (a) A distortion‐free GE reference for volunteer V2 with cumulative head rotation up to 7.8° performed during EPI acquisition compared with (b) the degree of distortion in raw EPI, (c) the accuracy of SDC, and (d) the accuracy of jittered‐TE DDC. Red lines highlight structures of interest (eg, brain boundaries, central sulcus). Distortions in SDC data reached up to 8.2 mm at the brain boundary (green arrows, fifth row). Unwarping with the jittered‐TE method left no residual distortions. Supporting Figure S2. (a) A distortion‐free GE reference for volunteer V4 with the cumulative head rotation up to 6.9° performed during EPI acquisition compared with (b) the degree of distortion in raw EPI, (c) the accuracy of SDC and (d) the accuracy of jittered‐TE DDC. Red lines highlight structures of interest (eg, brain boundaries, central sulcus). Distortions in SDC data reached up to 6.6 mm (green arrows, fifth row) and showed residual distortions of approximately 1.6 mm around the central sulcus (green arrows, sixth row). Unwarping with the jittered‐TE method left no residual distortions. Supporting Figure S3. Comparison of foot activation maps from volunteer V1 derived from standard (second row) and jittered‐TE (third row) EPI runs without distortion correction. The fourth row shows a manually defined anatomical ROI in the foot region of the primary motor cortex. Suprathreshold voxels from t maps in the anatomical ROI are shown in the fifth row for standard EPI and in the sixth row for jittered‐TE EPI. In the bottom row, mean BOLD signal change in suprathreshold voxels is plotted for standard (black) and jittered‐TE EPI (red), showing very similar behavior. [file MRM-76-1388-s001.docx]

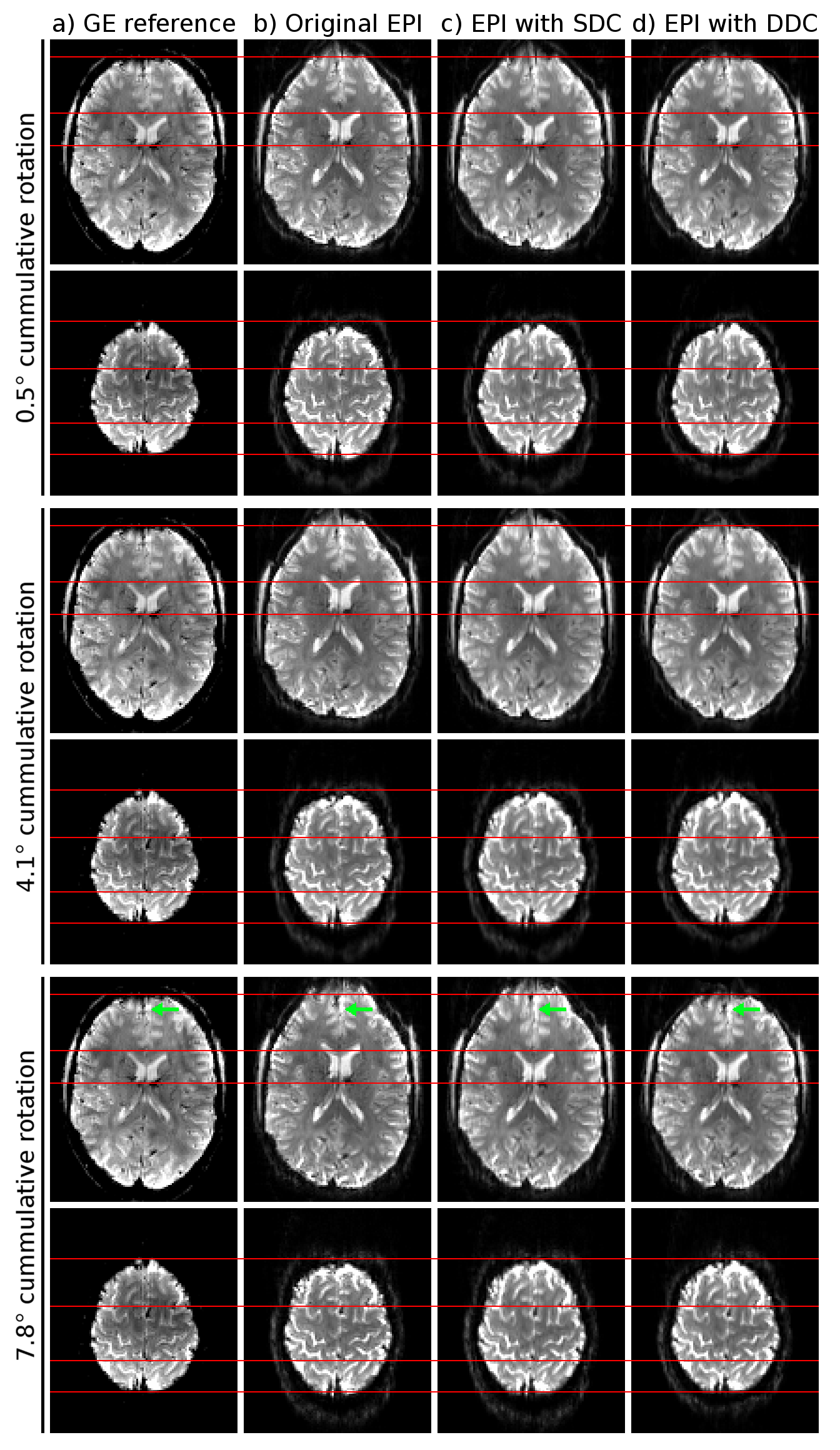


**FIG. S1.** The degree of distortion in raw EPI (**b)**, the accuracy of **c**: static distortion correction (SDC) and **d**: jittered-TE dynamic distortion correction (DDC) in comparison with **a**: a distortion-free GE reference for volunteer V2 with cumulative head rotation up to 7.8° performed during EPI acquisition. Red lines highlight structures of interest (e.g. brain boundaries, central sulcus). Distortions in SDC data reached up to 8.2 mm at the brain boundary (green arrows, 5^th^ row). Unwarping with the jittered-TE method left no residual distortions.


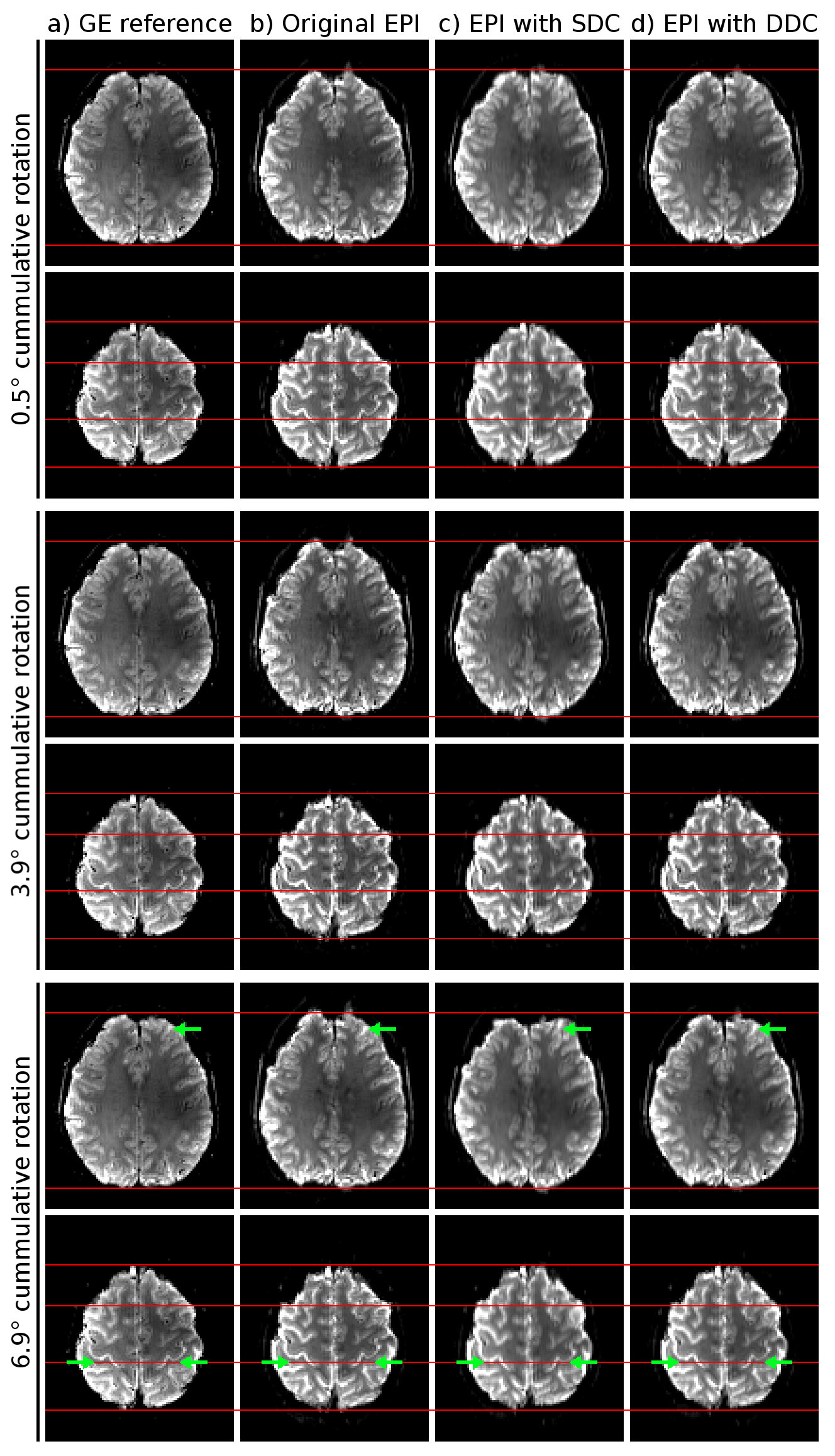


**FIG. S2.** The degree of distortion in raw EPI (**b)**, the accuracy of **c**: static distortion correction (SDC) and **d**: jittered-TE dynamic distortion correction (DDC) in comparison with **a**: a distortion-free GE reference for volunteer V4 with the cumulative head rotation up to 6.9° performed during EPI acquisition. Red lines highlight structures of interest (e.g. brain boundaries, central sulcus). Distortions in SDC data reached up to 6.6 mm (green arrows, 5^th^ row) and showed residual distortions of about 1.6 mm around the central sulcus (green arrows, 6^th^ row). Unwarping with the jittered-TE method left no residual distortions.


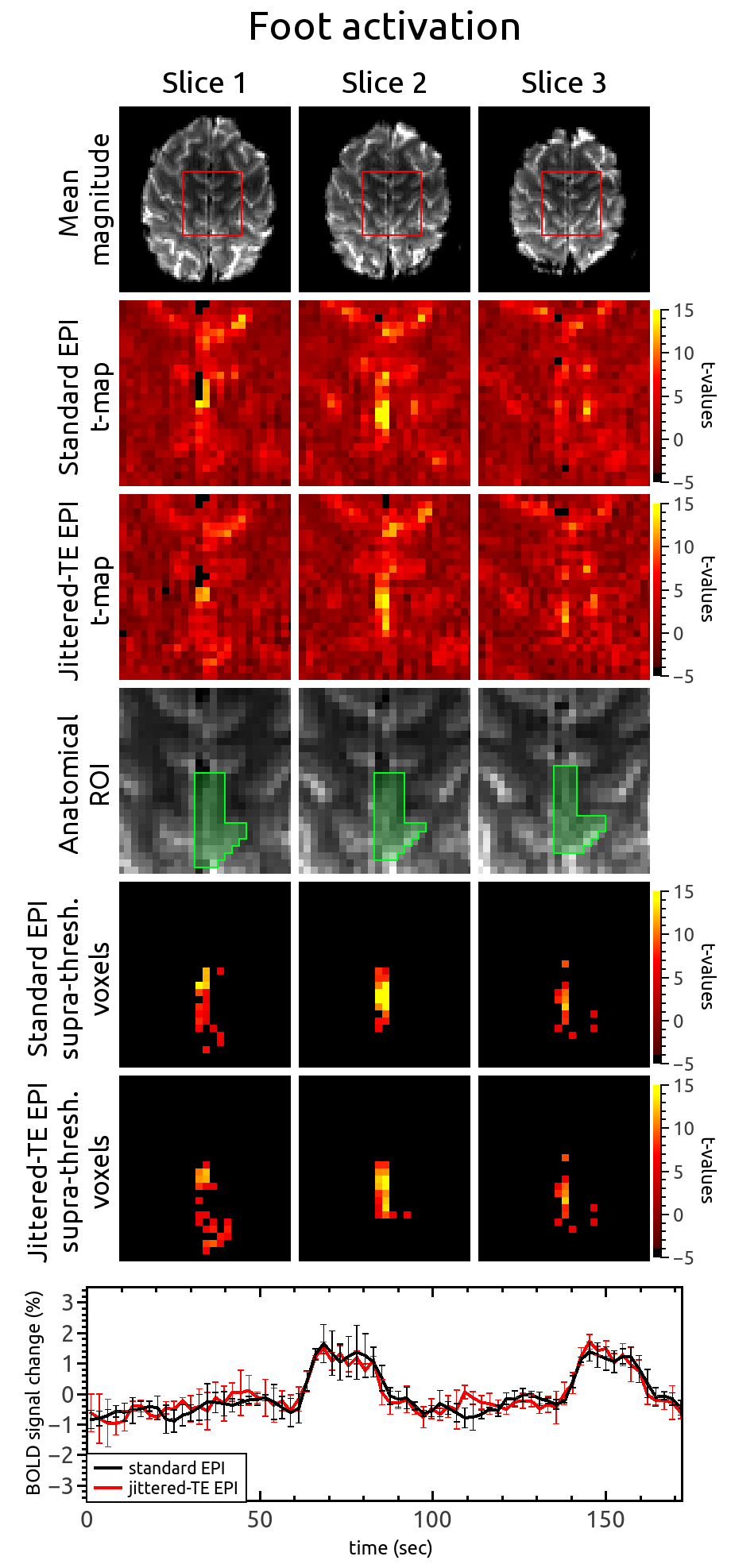


**FIG. S3.** Comparison of foot activation maps from volunteer V1 derived from standard (2nd row) and jittered-TE (3rd row) EPI runs without distortion correction. The 4th row shows a manually defined anatomical ROI in the foot region of the primary motor cortex. Supra-threshold voxels from t-maps in the anatomical ROI are shown in the 5th row for standard and in the 6th row for jittered-TE EPI. In the bottom row, mean BOLD signal change in supra-threshold voxels is plotted for standard (black) and jittered-TE EPI (red), showing very similar behavior.
